# A Study of the Evolution of Human microRNAs by Their Apparent Repression Effectiveness on Target Genes

**DOI:** 10.1371/journal.pone.0025034

**Published:** 2011-09-21

**Authors:** Yong Huang, Xun Gu

**Affiliations:** 1 Department of Genetics, Development, and Cell Biology, Center for Bioinformatics and Biological Statistics, Iowa State University, Ames, Iowa, United States of America; 2 School of Life Sciences, Center for Evolutionary Biology, Fudan University, Shanghai, China; South Texas Veterans Health Care System, United States of America

## Abstract

**Background:**

Even though the genomes of many model species have already been sequenced, our knowledge of gene regulation in evolution is still very limited. One big obstacle is that it is hard to predict the target genes of transcriptional factors accurately from sequences. In this respect, microRNAs (miRNAs) are different from transcriptional factors, as target genes of miRNAs can be readily predicted from sequences. This feature of miRNAs offers an unprecedented vantage point for evolutionary analysis of gene regulation.

**Methodology/Principal Findings:**

In this study, we analyzed a particular aspect of miRNA evolution, the differences in the “apparent repression effectiveness (*ARE*)” between human miRNAs of different conservational levels. *ARE* is a measure we designed to evaluate the repression effect of miRNAs on target genes based on publicly available gene expression data in normal tissues and miRNA targeting and expression data. We found that *ARE* values of more conserved miRNAs are significantly higher than those of less conserved miRNAs in general. We also found the gain in expression abundance and broadness of miRNAs in evolution contributed to the gain in *ARE*.

**Conclusions/Significance:**

The *ARE* measure quantifies the repressive effects of miRNAs and enables us to study the influences of many factors on miRNA-mediated repression, such as conservational levels and expression levels of miRNAs. The gain in *ARE* can be explained by the existence of a trend of miRNAs in evolution to effectively control more target genes, which is beneficial to the miRNAs but not necessarily to the organism at all times. Our results from miRNAs gave us an insight of the complex interplay between regulators and target genes in evolution.

## Introduction

Even though the genome sequences of many model species are available now, our knowledge of gene regulation in evolution, including the changes in the regulatory genes, the regulatory sequence elements, and their interactions, is still very limited. The reason is that it is still very hard to accurately predict targets of transcriptional factors from sequences. Current methods for predicting transcription factor binding sites, such as those described in [1], are not yet sufficiently accurate. Furthermore, transcriptional factors usually act cooperatively with other factors, but the prediction of protein-protein interaction alone is still an unsolved problem.

In contrast, the target genes of microRNAs (miRNAs) can be readily predicted from sequences. MiRNAs are small RNAs that are encoded in genomes with stable expression patterns in tissues. MiRNA-mediated gene regulation seems to have originated very early in evolution, possibly before the emergence of the common ancestor of eukaryocytes [2], [3]. They regulate a large number of target genes [4], [5], [6], in particular regulatory genes [7]. MiRNAs repress the expression of their target genes by base-pairing to target sites usually in the 3′ UTR of the transcripts of the genes. Predicting target sites (and thus target genes) of miRNAs is relatively straight forward. In plants, the target sites usually are almost perfectly complementary to the mature sequence of miRNAs [8], while in animals the target sites usually are complementary at least to the seed region of miRNAs [9], [10], [11]. *In vitro* experiments have shown that a single site alone is sufficient for miRNAs to exert the repressive effects [12]. While the predicted target genes of miRNAs still contain many false positives, their accuracy is much higher than that of the transcriptional factors. These features of miRNAs offer us an unprecedented vantage point to study their evolution and give us insights into the evolution of gene regulation.

Of particular interest is the difference in repression effectiveness among miRNAs of different conservational levels. It is well understood now that, due to the repressive effect of miRNAs on target genes, the 3′UTRs of highly expressed genes are less likely to have target sites of miRNAs expressed in the same tissues [12], [13], [14], [15]. On the other hand, phylogenetic studies have shown that miRNAs emerged in evolution progressively at time points matching major speciation events, such that the repertoire of miRNAs in an organism is a mixture of miRNAs of different conservational levels [16], [17]. MiRNAs have been described as paradoxical regulators for being both evolutionary conserved and functionally dispensable (Wu et al., 2009). Their effects on the expression of target genes are often found to be relative (Baek et al., 2008; Selbach et al., 2008), than absolute, like on-off switches (Bartel, 2009). We found that a lot of confusions have been caused in miRNA studies due to the lack of a quantitative measurement of the repression effects of miRNAs.

In this study, we designed the measure of “apparent repression effectiveness (*ARE*)” to quantify the repressive effect of miRNAs on the expression of target genes at transcript level. Using the *ARE* measuer, we found that more conserved miRNAs have significantly higher repression effectiveness on the target genes than less conserved miRNAs on average, which can be explained by the existence of an increasing trend in miRNA evolution to secure more target genes. The *ARE* measure results of miRNAs gave us an insight into the interplay between regulator and target genes in evolution and also opened many new possibilities for further study of the interplay in cell and evolution.

## Results

### Apparent repression effectiveness

The essential functionality of miRNAs is their repressive effect on the expression of target genes. However, this repressive effect is usually not complete. To quantify the repressive effect of miRNAs, we designed the “apparent repression effectiveness (*ARE*)” measure. As depicted in Table 1, for a particular miRNA in a tissue, genes are partitioned into target (*T*) and non-target genes (*N*), and into highly-expressed (*H*) and non-highly-expressed genes (*L*) (see Methods for detail). For a miRNA in a tissue, all the genes were partitioned into four categories, *TH*, *TL*, *NH* and *NL*. These symbols also denoted the number of genes in each category. The *ARE* of a miRNA in a tissue is the logarithm of the relative risk (RR) of finding genes highly expressed given not targeted versus targeted by the miRNA, which is expressed in the following formula, where the probabilities are calculated as frequencies from the data.
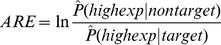
(1)

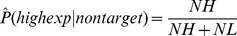
(2)

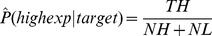
(3)The *ARE* is the logarithm of a relative risk (*RR*) statistic, so it has an approximate normal distribution when the numbers of genes in each partition in Table 1 is not overly small. The genes that have no target sites of any miRNAs were not included in this study, as they are not informative to the differences of *ARE* between miRNAs. The reason that *ARE* measures the “apparent” repression effectiveness is that the expression levels of genes used in this study are from normal tissues, which means they are posterior (after miRNA-mediated regulations) expression levels. *ARE* thus measures the combined reduction in gene expression caused by both miRNA-mediated degradation of mRNAs and endogenous mutual exclusiveness in expression patterns between miRNAs and target genes. Since miRNAs repress gene expression mainly by blocking translation in animals, mutual exclusiveness in expression patterns is expected to contribute substantially to *ARE*. A miRNA with a higher *ARE* value in a tissue is less likely to have highly-expressed target genes in the tissue. This will indicate that the miRNA has stronger influences on the expression of its target genes in the tissues.

**Table 1:** Tabulation of genes by expression levels and target sites, for *ARE* calculation.

*Number of genes*	*Expression level*	
	*High*	*Low*
***Non-target***	NH	NL
***Target***	TH	TL

Genes are divided into *TH* (target and highly expressed), *TL* (target and not highly expressed), *NH* (non-target and highly expressed) and *NL* (non-target and not highly expressed) categories for a miRNA and an expression cutoff. The symbols are also used to denote the numbers of genes in each category.

### *ARE* values of human miRNAs in tissues

We calculated the *ARE* values of human miRNAs with both expression and targeting information available in twelve tissues, including cerebellum, frontal cortex, heart, liver, prostate, uterus, thyroid, placenta, pancreas, testis, ovary, and pituitary. We used the genes expression profiles in human tissues from GNF atlas 2 [18]; human miRNA expression profile from the mammalian miRNA expression atlas [19]; and miRNA target prediction from PicTar [20]. In this study, all the gene expression data were at the transcript level.

**Figure 1:**
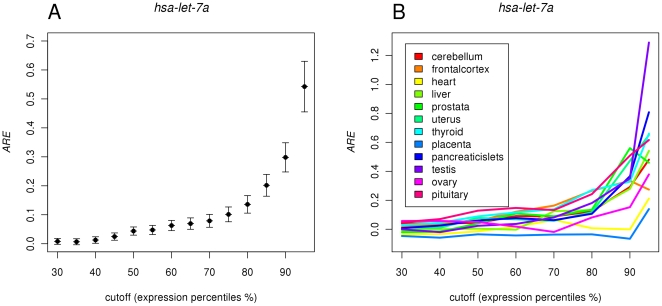
*ARE* values of a typical miRNA, *hsa-let-7a*, with different cutoffs for high expression. A) Means and standard errors of *ARE* values across tissues of *hsa-let-7a*, B) *ARE* values of *hsa-let-7a* in different tissues.

A miRNA was viewed to be expressed in a tissue if its clone count was greater than 0 from the mammalian miRNA expression atlas. The target genes of miRNAs were retrieved from the PicTar database [20]. For a chosen cutoff of high expression level, the numbers of genes in the *TH*, *TL*, *NH*, *NL* categories were counted and the *ARE* values were calculated as in Formula 1x1-37001r1. It should be noted that the choice of the expression cutoff is not trivial. Shown in Figure 1 are the *ARE* values for a typical miRNA, *hsa-let-7a* with different expression cutoff. The dots represent the mean *ARE* values across tissues where *hsa-let-7a* is expressed, and the error bars represent the standard error of the means. It is obvious from Figure 1 that *ARE* values are larger when the expression cutoff is set higher. This is expected, as miRNAs are negative regulators of gene expression and co-expression of miRNAs and highly expressed target genes are less likely to exist. It is also obvious that at relatively low expression cutoffs (such as 50%), the *ARE* values are very close to 0. This shows that, in general, the repressive effect of miRNAs on gene expression is more prominent at preventing their target genes from being highly expressed, than turning them off completely. It should be noted that, when the cutoff was set higher, the number of genes expressed above the cutoff was also smaller. As the result, the variances of the *ARE* values also became larger. In this study, we used 80% percentile as the cutoff for highly expressed genes, as the gene number above this cutoff is still big enough. Also, this is a commonly used cutoff in the literature.

Using the 80% percentile as the cutoff of high expression, the *ARE* values for each miRNA in each tissue were calculated and listed in Table S1. The *ARE* values in the tissues where the miRNA was not expressed were also calculated for control purposes. It is obvious from Figure 1B that *ARE* values of the same miRNA differ greatly in different tissues where the miRNAs is expressed. This is not unexpected, as not only the targeting and expression information of miRNAs is not yet perfectly accurate, but the expression levels of miRNAs also differ greatly among tissues. One representative example is the *hsa-miR-1* case, as is shown in Table 2. The *ARE* value of *hsa-miR-1* is 0.2961 in heart, where the miRNA is highly expressed (clone count 15). However, in tissues of prostate, uterus, thyroid, testis, and ovary where *hsa-miR-1* is expressed at very low levels (clone counts 1 or 2), the *ARE* values are small and negative. The *ARE* values calculated from tissues where the miRNAs are highly expressed are more reliable, considering the potential errors in miRNA expression profiling (some miRNAs with clone count 1 or 2 may not be really expressed in the tissue). In this regard, for each miRNA, we chose the *ARE* value from the tissue where the miRNA was most highly expressed (take median in case of tie) as the representative *ARE* (*rARE*) of the miRNA and used it for comparisons between miRNAs.

**Table 2:** Tissue differences in *ARE* values of *hsa-miR-1*.

*Tissue*	*ARE*	*miRNA expression (clone count)*
***Heart***	0.2961	15
***Prostate***	−0.2381	1
***Uterus***	−0.2598	1
***Thyroid***	−0.2282	2
***Testis***	−0.0245	1
***Ovary***	−0.1177	1

The *ARE* value in the tissues where a miRNA is most highly expressed is denoted as *rARE* (use median in case of tie). For *hsa-miR-1* in the tissues where it was expressed, the *ARE* values were listed below. For *hsa-miR-1*, the *rARE* value is the *ARE* value from heart.

### Differences in *rARE* values among human miRNAs of different conservational categories

We compared the *rARE* (representative *ARE*) values among human miRNAs of different conservational categories. We classified human miRNAs into three categories based on the presence of their homologs in metazoan species, as was described in our previous report [17]. The phylogenetic distribution of miRNAs was based on the miFam feature from miRBase. Briefly, a miRNA was classified into category I if it has homologs in mammals, non-mammal vertebrates, and invertebrates; category II if it has homologs only in mammals and non-mammal vertebrates; and to category III if it has homologs only in mammals (see Methods for details). We used only miRNAs with both targeting and expression information available in this study, which covered 38 human miRNAs in category I, 76 in category II and 19 in category III. All the miRNAs are conserved at least between human and mouse. The calculated *rARE* values and the conservational category classification of miRNAs are summarized in Table S2.

**Figure 2:**
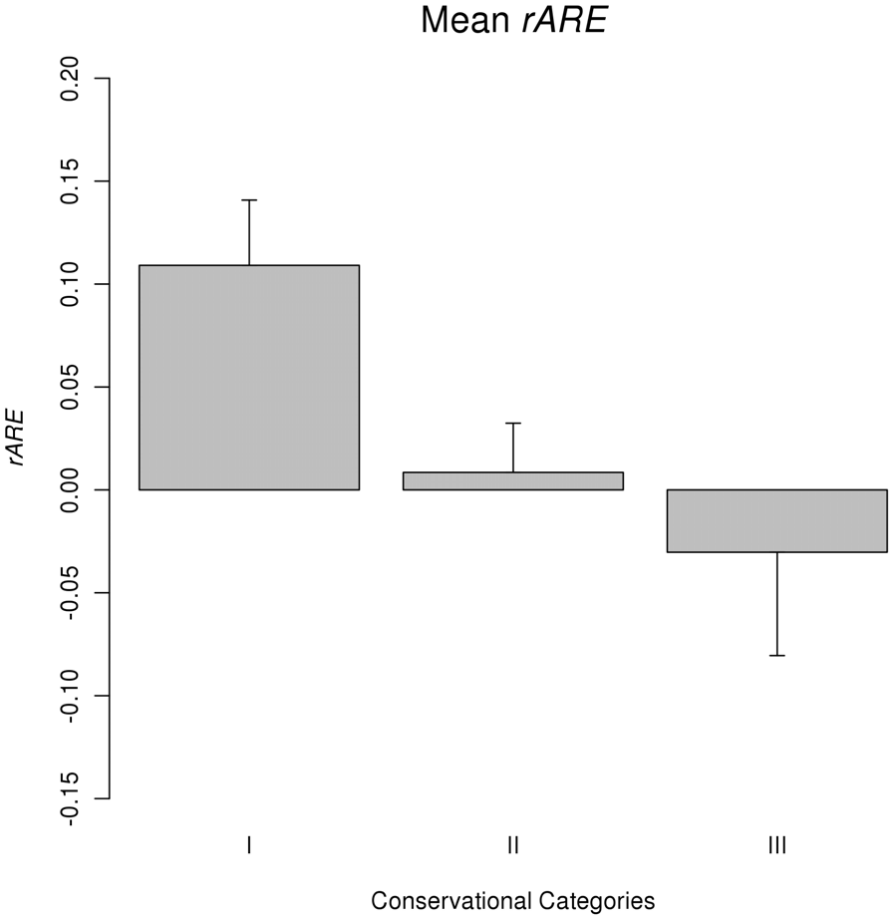
Differences in *rARE* for miRNAs in different conservational categories. The most conserved miRNAs in Category I has significantly higher *rARE* values than miRNAs in less conserved categories.

The comparison of the *rARE* values among miRNAs showed an interesting trend among different conservational categories. As is shown in Figure 2, the mean *rARE* values of miRNAs is the highest in the most conserved Category I and lowest in the least conserved Category III (see Table 3 for values of means and standard errors at cutoff 80%). We carried out ANOVA, using the *rARE* values as the response variable and conservational categories as the explanatory variable. The result showed that the conservational category is a significant factor for *rARE* values of miRNAs (F-test p-value = 0.0203). Comparison of the mean *rARE* values using Tukey's HSD test showed that the mean *rARE* value of Category I miRNAs is significantly larger than that of Category II (p-value = 0.0404) and Category III (p-value = 0.0459). The difference between Category II and III is not significant by Tukey's HSD test, likely resulted from the large standard error of mean *rARE* value in Category III (which has a small number of miRNAs).

**Table 3:** Mean *rARE* and standard error for miRNAs in different conservational categories under different cutoffs of high expression.

*Cutoff*	*Category I*	*Category II*	*Category III*	*p-value*
**60%**	0.0533±0.0223	−0.0082±0.0119	−0.0850±0.0376	<0.001
**70%**	0.0794±0.0275	0.0024±0.0155	−0.1080±0.0469	<0.001
**80%**	0.1091±0.0317	0.0085±0.0239	−0.0303±0.0502	0.0203
**90%**	0.2445±0.0637	0.0489±0.0225	0.0079±0.0670	<0.001

The differences in *rARE* among the miRNA conservational categories were found to be significant with different expression cutoffs (60–90%) tested.

The finding of more conserved miRNAs having higher *rARE* values than less conserved miRNAs on average suggested the existence of an increasing trend in miRNAs to effectively control more target genes in evolution. It has been reported that the birth and death of new miRNAs happen frequently in evolution [21]. In this respect, our result can be explained that in miRNA evolution, there may be continuous pressure for the miRNAs to secure enough target genes to ensure its own existence in the genome.

### Control analyses

We carried out several control analyses to confirm that the finding of the increasing trend in *rARE* values among miRNAs of different conservational categories was not caused by chances or by the use of a particular set of data.

First, since the cutoff of high expression was arbitrarily set at 80% percentile in the previous section, we tested whether using a different cutoff might change the results. We tested different cutoff values including 60%, 70%,, 80% and 90%. The results are summarized in Table 3. We can see that the increasing trend in the mean *rARE* values in miRNAs of different conservational categories exists at all the expression cutoffs. At all the cutoff values, the p-values for the significance of the conservational category as a factor are smaller than 0.05. The p-value at 80% is actually the least significant compared to other cutoffs. Meanwhile, similar to the example of *hsa-let-7a* in Figure 1A, the mean *rARE* values are generally higher under higher expression cutoff. This is in accordance with the common wisdom that it is less likely to find highly expressed target genes in the same tissue with the miRNA. The standard errors also increase as the cutoffs increases. This is due to the smaller number of genes expressed above higher cutoffs. Taken together, the results are generally consistent under different cutoffs.

We also examined the *ARE* values in the tissues where the miRNAs were not expressed. For a miRNA, the mean of the *ARE* values in these tissues without expression was used to compare among different conservational categories. One complication here was that, in a tissue where a miRNA was not expressed, its target genes might still be targeted by other miRNAs expressed in that tissue. In this regard, for the control analysis, we limited the non-target genes (in NH and NL categories) of a miRNA in a tissue to be those that have no target sites of any other miRNAs that were expressed in the tissue. The results are shown in Table 4. It is clear that the *ARE* values are very low (below −0.09 in all categories). There is also not a significant difference between different conservational categories (F-test p-value = 0.5646). This analysis showed that the trend we observed in Figure 2 is not by chance.

**Table 4:** Mean *ARE* values and standard errors of miRNAs in tissues without expression.

*Category I*	*Category II*	*Category III*	*p-value*
−0.1739±0.0826	−0.0918±0.0578	−0.1856±0.0822	0.5646

As a control, mean *ARE* values were calculated in the tissues where a miRNA was not expressed. No significant differences in these mean *ARE* values was observed among the miRNAs, showing the differences in *rARE* is not caused by chance.

We further analyzed the *rARE* values using a different set of predicted targets from TargetScan Human [11]. All the other settings were the same, only the predicted targets were from TargetScan, instead of PicTar. The results are shown in Table 5. A similar trend in the *rARE* values is observed, with the more conserved miRNAs having higher *rARE* values on average. The result is consistent with those based on the PicTar set of predicted targets. One concern with the TargetScan set of target for this analysis was that the predicted targets in TargetScan were filtered by inter-species conservation. For the PicTar set this has not been a problem, since site conservation is only among mammal species in PicTar and all the miRNAs in this study are conserved at least between human and mouse. However, site conservation is more strictly requested to be among mammals and chicken in TargetScan. As the result, some bias may exist in Category III as some true targets of miRNAs from Category III may not be included in the TargetScan set. However, these differences in the target sets have not influenced the general trend we observed in the *rARE* values.

**Table 5:** Mean *rARE* values and standard error for miRNAs based on TargetScan set of miRNA targets.

*Category I*	*Category II*	*Category III*	*p-value*
0.2090±0.0717	0.0544±0.0216	−0.0241±0.0363	<0.001

Using a different set of predicted targets of miRNAs from TargetScan resulted in the same conclusion that more conserved miRNAs have higher *rARE* on average.

### Covariates that may also influence *ARE*

We have shown that the conservational category of miRNAs to be a significant factor for the differences in *rARE* values among the miRNAs. We further analyzed several covariates that might influence *ARE* more directly, including expression abundance (as clone counts) and broadness (as number of tissues with expression) of miRNA, and the accessibility of target sites.

**Figure 3:**
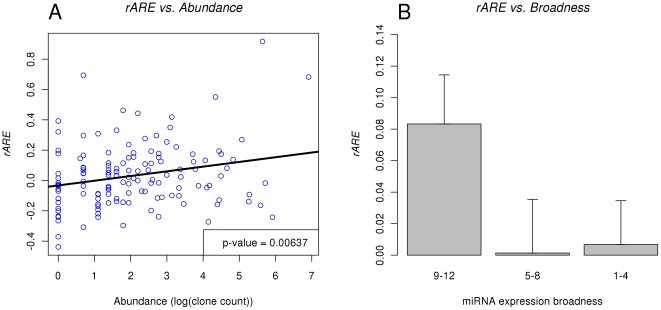
Influence of expression abundance and broadness on *rARE*. A) Regression of *rARE* on expression abundance of miRNAs (as log(clone count)), B) Mean *rARE* values for miRNAs of different expression broadness (as number of tissues).

**Figure 4:**
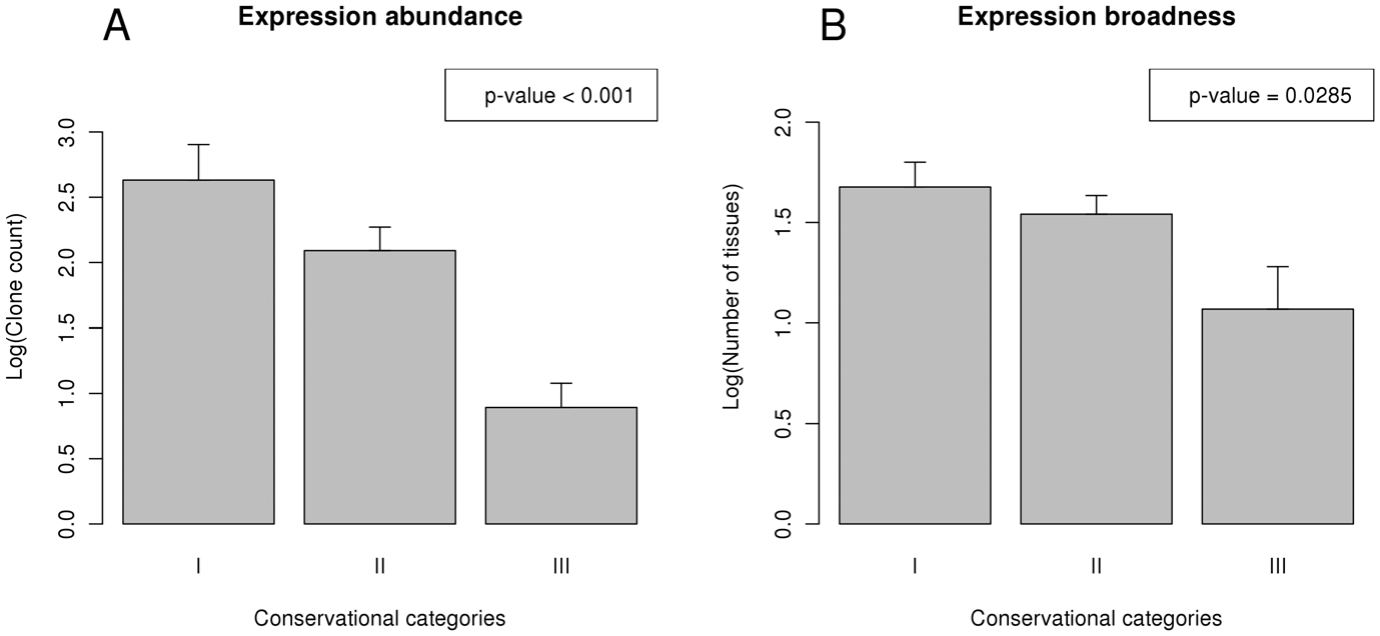
Differences in expression abundance and broadness among miRNAs in different conservational categories. A) Difference in expression abundance (in tissue with maximal expression) among miRNAs in different conservational categories, B) Difference in expression broadness among miRNAs of different conservational categories.

In Figure 3A, we plotted the *rARE* values of miRNAs against the expression abundance (as logarithm of clone counts) of the miRNAs in the tissues where the *rARE* values were calculated from. Using the *rARE* values of miRNAs as the response variable and expression abundance as explanatory variable, an ANOVA analysis showed that expression abundance to be a significant factor for *rARE* values (F-test p-value = 0.006373). It is obvious that the expression abundance of miRNAs has a significant effect on the *rARE* values, but it should also be noted that the variance of *rARE* is also large. In Figure 3B, we calculated the mean *rARE* values for miRNAs of different expression broadness (as number of tissues with expression). It is also obvious that miRNAs that have broad expression pattern (expressed in more than 8 tissues) have a mean *rARE* value much greater than miRNAs expressed in 8 or less tissues. The standard error of the mean of *rARE* in Figure 3B is also large. These results show that in tissues where a miRNA was highly expressed or if a miRNA was broadly expressed in tissues, the miRNA was more likely to have high *rARE* values. Both expression abundance and broadness of miRNAs are significantly different among miRNAs of different conservational categories, as is shown in Figure 4 (the p-values are from the F-test with conservational category as the explanatory factor). However, the variance of *rARE* for miRNAs with similar expression abundance or broadness is also large, suggesting many other factors may also be in play.

**Figure 5:**
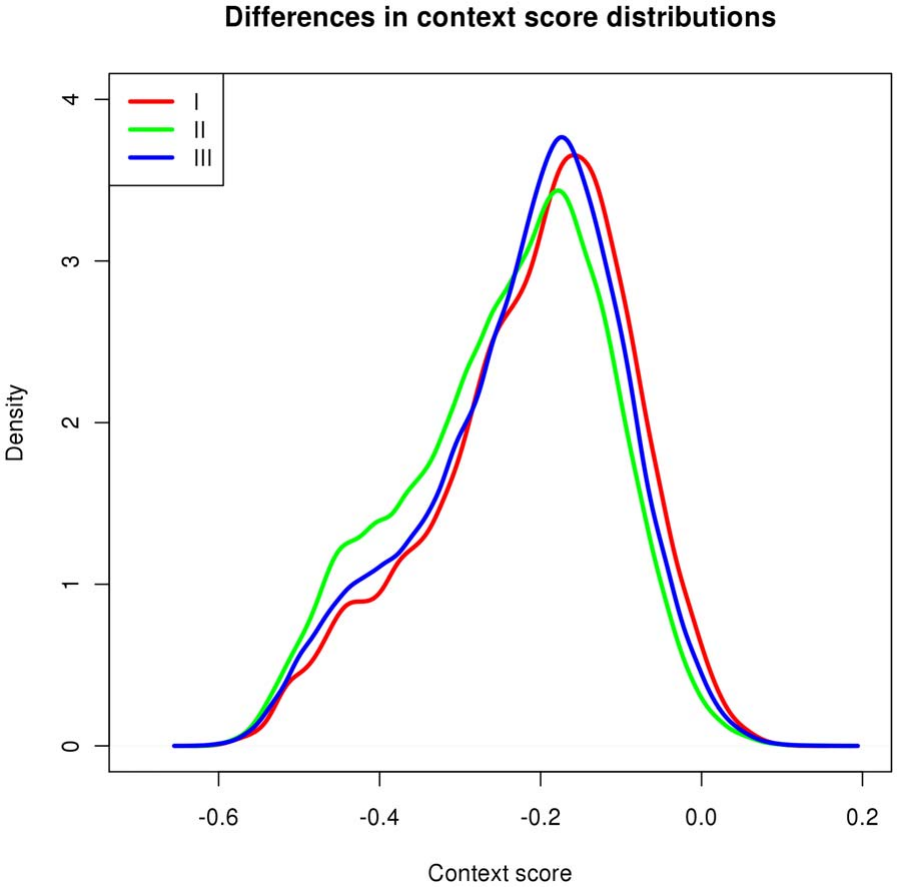
Distribution of context scores of sites of miRNAs in different conservational categories. No significant difference in the distribution of context scores is observed among target sites of miRNAs of different conservational categories.

The accessibility of target site has also been reported to be an important factor for miRNA-mediated regulation [22]. It has been suggested that deeply conserved miRNAs may have sites that are more accessible [5], [23]. The TargetScan set of predicted miRNAs site has the “context score” feature integrating information based on 3′ paring, local AU content and distance from UTR ends of miRNA target sites, which servers as a measure of the accessibility of miRNA target sites [10]. In this regard, we examined the differences in context score for sites of miRNAs in different conservational categories. The results are summarized in Figure 5. Different from what has been suggested in other reports, we found the distributions of the context score of target sites are almost identical for miRNAs in different conservational categories. It should be noted that the target sites we used in this study are the conserved target sites, as they are generally believed to be more reliable. In general, at least for the conserved target sites, the context score does not seem to differ greatly among miRNAs from different conservational categories. This may suggest that the evolution of the accessibility of target sites was fast in evolution, such that all target sites that are conserved in species have similar distribution in their accessibility.

### Interactions between miRNAs

**Figure 6:**
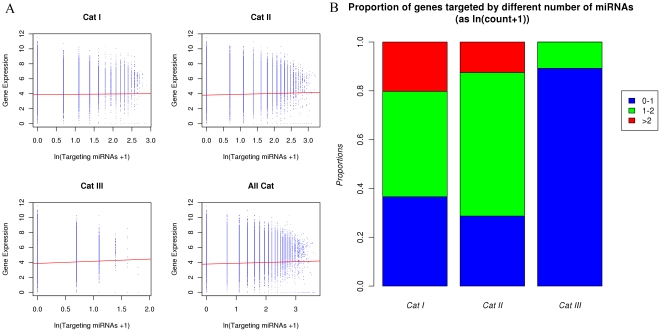
Interacting of miRNAs in targeting. A) Gene expression levels were plotted against the number of targeting miRNAs (as ln(Targeting miRNAs+1)), for miRNAs in each category or all together. The red line in each graph was the linear regression line based on the data points. B) For each miRNA category, the proportion of genes with ln(Targeting miRNAs+1) in 0–1, 1–2 or >2 were shown. Category I miRNAs have more interaction in targeting compared to other miRNAs.

Another concern for this study was that how might the interaction between different miRNAs targeting the same gene influence our conclusions. We further analyzed the composition of targeting miRNAs for each gene. In Figure 6A, we plotted the gene expression levels (normalized at array level, from [18]) against the number of targeting miRNAs (each targeting miRNA count only once, even if there are multiple target sites) in each conservational categories and all categories combined. While the linear regression line in all four plots are almost all flat, it was interesting to find that genes that were targeted by larger number of miRNAs have smaller variance in expression. This is obviously seen in Figure 6A, as all the plots have a “>” shape. The pattern suggested that genes that are heavily targeted by many miRNAs tend to have a expression at medium level, which is in accordance to the canalization view of miRNA function [24]. While this pattern is identical in all miRNA conservational categories, the distribution of the number of targeting miRNAs is different between the categories, as is shown in Figure 6B. We can see that the proportion of genes that are targeted by more than 6 (ln(count+1)>2) miRNAs are higher for Category I miRNAs than that for Category II and III miRNAs. Similar results were found with TargetScan data, which can be found in Figure S1.

This result suggested a mechanism of how interaction in miRNA targeting may contribute to the repression effectiveness of miRNAs. While genes targeted by many miRNAs tend to be expressed at medium level, more conserved miRNAs also tend to target common genes. As the result, genes targeted by more conserved miRNAs were less likely to be expressed at high levels. It should be noted that *ARE* measures the marginal property of miRNA-mediated repression, which concerns only what proportion of target gene were repressed given a miRNA was expressed in a tissue. This is a necessary simplification, as very few other regulation details of the genes are reliably understood now.

## Discussion

In this study, we designed the apparent repression effectiveness (*ARE*) measure to quantify the repressive effects of miRNAs on the expression of their target genes. We used the representative *ARE* (*rARE*) for comparison between miRNAs. An increasing trend in *rARE* was observed, in which more conserved miRNAs were found to have higher *rARE* in general. The results were confirmed by control analyses using different data sets and cutoffs.

It should be noted that the conservational level of miRNAs is an associated factor rather than a direct causative factor for the *ARE* of the miRNAs. For individual miRNAs in particular tissues, the *ARE* value may be influenced by more direct factors such as co-expressed miRNAs targeting the same mRNA, or the type and number of binding sites. What our results showed was that, on average, more conserved miRNAs have higher *rARE* values. Many evolutionary processes may have contributed to this outcome, such as the gain of stable expression pattern for conserved miRNAs in evolution and the co-evolution of genes and miRNAs to achieve mutual exclusiveness in expression.

It should also be noted that only the human miRNAs that are conserved at least in mouse, and have both expression and targeting information, were used in this study. While these miRNAs cover most of the classical miRNAs they still represented just a subset of all human miRNAs. Large number of new miRNAs were being added to the public database continuously, many of which were lineage specific. Expression and targeting information of the new miRNAs are mostly not available yet. Further analyses with a more complete set of human miRNAs can help to confirm the generality of the findings in this study, when data become available.

Our *ARE* results gave us an insight into the interplay between regulators and target genes in evolution. There are recent reports showing that lowly expressed miRNAs (mostly lineage specific miRNAs) evolve fast [25], and lineage specific miRNAs exert adverse effects when expressed in different species [26]. Furthermore, more conserved miRNAs were generally found to be expressed more broadly and target more miRNAs [23]. All these findings are consistent with our results in suggesting a simple model for miRNA evolution, in which there is a “selfish” tendency in miRNAs to secure more target genes in evolution. The “selfish” tendency is beneficial for the existence of the miRNAs in the species, but is not necessarily beneficial to the organism. However, the “selfish” tendency of miRNAs helped to provide more choices of gene regulation for selection in evolution, which may be critical to the organisms at the time of major speciation.

From many aspects, miRNA-mediated gene regulation is much simpler than that by transcriptional factors and lends itself to evolutionary studies. We have focused on miRNAs in this study. The insights we gained of gene regulation evolution from miRNA studies will eventually help us to understand the more complicated evolution of transcription factor mediated regulation when more data become available.

## Materials and Methods

### Calculating *ARE* values

For calculating the *ARE* value of a miRNA in a tissue, all the genes were partitioned into target (*T*) and non-target genes (*N*), based on whether a gene contained at least one target sites of the miRNA (based on the miRNA target prediction in use, PicTar or TargetScan in this study). The genes were also partitioned into highly-expressed (*H*) and non-highly-expressed genes (*L*), based on whether a gene has expression level above (including equal to) or below the cutoff in the tissue. The expression cutoff was chosen to be the 80% percentile (higher percentile for higher expression) of all the genes in a tissue, based on GNF atlas 2 (see below). The *ARE* value for a miRNA in a tissue was then calculated as described in Table 1 and Formula 1.

### MiRNA sequences and phylogeny

The sequences of miRNAs were retrieved from miRBase (Release 12) [27]. The phylogenetic distribution of miRNAs is based on the miFam feature from miRBase. We classified the human miRNAs, on a family basis, into three conservation categories base on the presence of their homologs in metazoan species [17]. If a miRNA family contains homologs in mammals, non-mammal vertebrates, and invertebrates, it is in category I; if a miRNA family contains homologs only in mammals and non-mammal vertebrates, it is in category II; if a miRNA family contains homologs only in mammals, it is in category III. This categorization offered us a basic estimation of the evolutionary age (time since emergence) of miRNAs. We used only miRNAs with both targeting and expression information available in this study, which covered 38 human miRNAs in category I, 76 in category II and 19 in category III.

### MiRNA expression profiles

We used the miRNA expression data of the mammalian miRNA expression atlas [19], which was one of the most comprehensive miRNA expression profiles available. The data were based on 256 small RNA libraries from 26 different organ systems and cell types in human and rodents. In our analysis, we used data from the 12 human tissues where gene expression data are also available. These tissues were cerebellum, frontal cortex, heart, liver, prostate, uterus, thyroid, placenta, pancreas, testis, ovary, and pituitary.

In this data set, the cloning frequencies of miRNAs were used as the measure of miRNA expression. In total, the data set covered 340 human miRNAs (by mature form), encoded by 395 miRNA genes (expression levels divided evenly between precursors that produce the same mature forms) from 214 transcript units.

### Human gene expression data

Gene expression profiles in human were retrieved from the GNF expression atlas 2 dataset [18]. A cutoff of 200 was applied to the raw readings to control noise. The expression data were already normalized at array level. To normalize between different tissues, we use a percentile-based approach. In each tissue, the percentiles (from 10% to 95% step by 5%) of gene expression levels were calculated and used in the analysis.

### MiRNA target site prediction

The predicted miRNA target sites were retrieved from PicTar [20]. We used the set of human miRNA targets in which the sites were conserved among five mammals (human, chimp, mouse, rat and dog) (the “Lall et al. 2006” set). The TargetScan (TS) set of targets were used as control. It was retrieved from TargetScan [11], Release 5.0. The targets in the TargetScan were conserved between human, mouse, rat, dog and chicken.

### Statistical analysis

ANOVA and regression analyses were carried out using R (http://www.r-project.org/). Raw data and source codes are available upon request.

## Supporting Information

Figure S1 **Interacting of miRNAs in targeting based on TargetScan data.** This shows a similar pattern to that in Figure 6 of the interacting of miRNAs in targeting is also found using TargetScan data.
(TIF)

Table S1 **Full list of** ***ARE*** **values of all the miRNAs studied.** Included in the table are the miRNA names, the tissue names, the expression levels of the miRNA in the tissues (based on [19]), the calculated *ARE* values, and the conservational categories of the miRNAs.
(XLS)

Table S2 **Full list of** ***rARE*** **values of miRNAs.** Included in the table are the miRNA names, the median *ARE* values of miRNAs in all the tissues, the *rARE* value, the expression broadness of the miRNAs, and the maximal expression levels of miRNAs in the tissues.
(XLS)
