# Supplementary figures and images for: A Study of the Evolution of Human microRNAs by Their Apparent Repression Effectiveness on Target Genes
Source: PLoS One. 2011 Sep 21;6(9):e25034. doi: 10.1371/journal.pone.0025034 (PMC3177854; doi:10.1371/journal.pone.0025034)

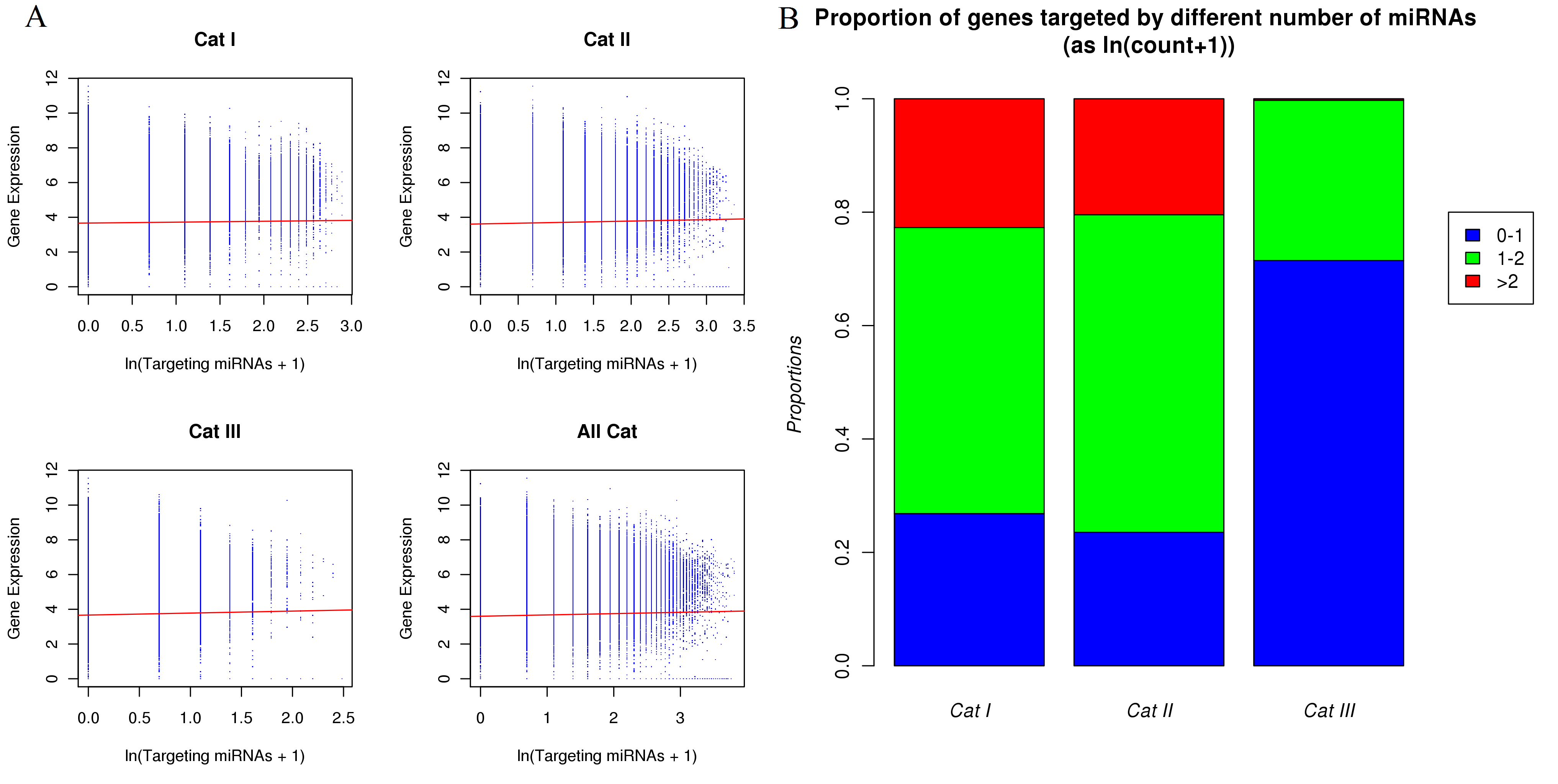

Supplement: Figure S1 — Interacting of miRNAs in targeting based on TargetScan data. This shows a similar pattern to that in Figure 6 of the interacting of miRNAs in targeting is also found using TargetScan data. (TIF) [file pone.0025034.s001.tif]
